# Phase I study of BC001, a novel fully human immunoglobulin G1 monoclonal antibody targeting the vascular endothelial growth factor receptor‐2, in advanced solid tumors

**DOI:** 10.1002/cam4.70208

**Published:** 2024-11-19

**Authors:** Dan Liu, Jifang Gong, Muling Liu, Huan Zhou, Shumei Wang, Jian Yang, Chenwei Shang, Xinlei Guo, Cha Wang, Yanqiao Zhang, Lin Shen

**Affiliations:** ^1^ Key Laboratory of Carcinogenesis and Translational Research (Ministry of Education), Early Drug Development Center Peking University Cancer Hospital & Institute Beijing China; ^2^ Key Laboratory of Carcinogenesis and Translational Research (Ministry of Education), Department of Gastrointestinal Oncology/Early Drug Development Center Peking University Cancer Hospital & Institute Beijing China; ^3^ Department of Gastrointestinal Surgery The First Affiliated Hospital of Bengbu Medical College Bengbu China; ^4^ Office of Drug Clinical Trial Facility The First Affiliated Hospital of Bengbu Medical College Bengbu China; ^5^ Phase I Clinical Trial Research Center Cangzhou City People's Hospital Cangzhou China; ^6^ Department of Radiotherapy Cangzhou City People's Hospital Cangzhou China; ^7^ Sichuan Luzhou Buchang Bio‐Pharmaceutical Co., Ltd Luzhou China; ^8^ Department of Gastrointestinal Medical Oncology Affiliated Cancer Hospital of Harbin Medical University Harbin China; ^9^ State Key Laboratory of Holistic Integrative Management of Gastrointestinal Cancers, Beijing Key Laboratory of Carcinogenesis and Translational Research, Department of Gastrointestinal Oncology Peking University Cancer Hospital & Institute Beijing China

**Keywords:** gastric cancer, phase 1 study, solid tumor, target therapy, vascular endothelial growth factor receptor‐2

## Abstract

**Background:**

BC001 is a novel fully human immunoglobulin G1 monoclonal antibody blocking VEGFR2. This phase I study aimed to assess BC001 alone and plus chemotherapy in solid tumors.

**Methods:**

In dose escalation part, BC001 was assessed at dose levels of 2, 4, 8, 12, 16 mg/kg on day 1,15, every 28 days, followed an accelerated titration and “3 + 3” design. BC001 plus paclitaxel was assessed at dose level of 8, 10 mg/kg. The primary endpoints included the DLT, MTD and RDE of BC001. In dose expansion part, it aimed to assess the anti‐tumor activity of BC001 alone and plus chemotherapy in gastric cancer as 2nd line treatment.

**Results:**

Overall, 53 patients were finally enrolled in this study (phase Ia, *n* = 28; phase Ib, *n* = 25). In phase Ia part, 1 DLT (grade 4 neutropenia lasting 4 days) was observed in BC001(8 mg/kg) plus paclitaxel cohort. MTD was not reached. All patients experienced TEAEs of any grade. Twenty‐four of them suffered ≥ grade 3 TEAEs. leukopenia (*n* = 33, 62.3%), hemoglobin decreased (*n* = 32, 60.4%), neutropenia (*n* = 29, 54.7%) were commonly observed hematological toxicities. The half‐life of 140‐240 h. And the PK parameters were not largely influenced by combination with paclitaxel. The serum sVEGFR‐1, VEGF‐A, sVEGFR‐2 could not predict the efficacy. Based on the safety, PK and efficacy data, BC001 of 8 mg/kg was determined as RED. Among the GC patients(*n* = 21) who receiving BC001 plus paclitaxel as 2nd‐line treatment in phase 1b part, 6 patients achieved PR and 10 patients experienced SD. The ORR was 28.6% (95% CI 11.3%, 52.2%) and the DCR was 76.2% (95% CI 52.8%, 91.8%), with the median PFS of 5.4 months (95% CI 1.9, 7.0) and OS of 9.4 months (95% CI 5.4, NA). The median DoR was 5.1 months (95% CI 2.6, NA).

**Conclusions:**

BC001 showed acceptable safety profile and preliminary response in both single‐agent and combination with chemotherapy cohorts, especially in GC.

## 1 INTRODUCTION

The VEGF (vascular endothelial growth factor)/VEGF receptor‐2 (VEGFR‐2) dependent pathway was a critical pathway involved in tumor angiogenesis and lymphangiogenesis, which contributed to tumor genesis and progression.^1^ Blocking VEGFR2 signaling by anti‐VEGF‐A or VEGFR‐2 antibodies or VEGFR small‐molecule tyrosine kinase inhibitors showed confirmed anti‐tumor activity in both preclinical and clinical practice.^1^, ^2^, ^3^ Up to now, ramucirumab, a fully human IgG1 antibody targeting VEGFR2 and VEGF‐A, was the only ant‐VEGFR2 antibody approved in China for gastric cancer.^3^ However, there were several other non‐gastric cancer, such as colorectal cancer, ovarian cancer, primary peritoneal, or fallopian tube cancer, neuroendocrine tumor, showing tumor response to anti‐VEGFR2 antibodies.^4^, ^5^, ^6^ Few anti‐VEGFR2 antibodies has been further evaluated in these non‐gastric tumors. Ramucirumab combined with paclitaxel was not approved in China for the treatment of gastric or gastroesophageal junction adenocarcinoma until March 2022. Before this approval, no anti‐angiogenic antibodies were available for gastric cancer or other solid tumors treatment in China, highlighting the significant clinical need and large market potential for VEGFR2 antibodies in the country.

BC001 is a novel engineered fully human immunoglobulin G1 (IgG1) mono‐clonal antibody targeting the VEGFR2, with decreased the likelihood of anti‐drug antibodies (ADAs) and allergic reactions. It was independently researched and developed in China. In vitro, it could specifically bind to VEGFR2, inhibit VEGF‐stimulated endothelial cell migration, tube formation, and effectively suppressed the trans‐differentiation of cancer stem cells into endothelial cells. The preclinical study, including a laser‐induced choroidal neovascularization (CNV) model in rhesus monkeys, demonstrated BC001's ocular efficacy with significant reduction in fluorescein leakage and retinal disruptions. Additionally, its antitumor efficacy was validated in various cancer models, such as human colorectal, gastric cancer, hepatocellular carcinoma xenografts in mice, where BC001 substantially inhibited tumor growth and weight.^7^, ^8^, ^9^ And in mice PDX models, it showed that the drug activity and toxicity profiles are comparable with ramucirumab, an existing VEGFR2 IgG1 monoclonal antibody that showed confirmed antitumor activity (Figure S1).

This study was designed to evaluate the safety and determine the RED of BC001 alone as well as BC001 plus paclitaxel for patients with advanced solid tumors in dose escalation (phase Ia) part. It also assessed the preliminary anti‐tumor activity and survival of BC001 alone as well as BC001 plus paclitaxel in selected tumor cohorts in dose expansion (phase Ib) part.

## 2 MATERIALS AND METHODS

### 2.1 Study design and treatment

This is a multicenter, open‐label, two‐part, phase I trial of BC001 done at 4 hospitals in China (CTR20181073/ChiCTR2300069367) from February, 2019 to October, 2022. The study was initiated and sponsored by Sichuan Luzhou Buchang Bio‐Pharmaceutical Co., Ltd. The protocol was approved by the ethics committees of all centers. This study was performed in accordance with the International Conference on Harmonization Guidelines for Good Clinical Practice and the Declaration of Helsinki. All eligible patients provided written informed consent.

This study was composed of two parts: a dose‐escalation in advanced solid tumors (phase Ia) and dose‐expansion phase in gastric cancer (phase Ib). The design was detailed in Figure S2. The dose design for BC001 monotherapy is based on the NOAEL (No‐Observed‐Adverse‐Effect Level) guidelines for estimating the Maximum Recommended Starting Dose (MRSD) for initial clinical trials. Five dose levels (2 mg/kg, 4 mg/kg, 8 mg/kg, 12 mg/kg, 16 mg/kg) have been designed for the clinical trial. Phase Ia part was a dose defining phase that initiated by an accelerated titration design at BC001 2 and 4 mg/kg on day 1,15, every 28 days, in which only 1 patient was required if none of grade ≥2 treatment‐emergent adverse events (TEAEs) related to study drug occurred during the 28‐day tolerability observation after first infusion. Otherwise, additional two or five patients were needed. Standard 3 + 3 escalation scheme was adopted thereafter, at dose level of 8,12,16 mg/kg on day 1,15, every 28 days. The dose level of 16 mg/kg was designed to enroll 6 patients. At dose level of 8 mg/kg and 10 mg/kg, BC001 was combined with paclitaxel (80 mg/m^2^ d1,8,15, q28d) in 6 patients to explore the recommended dose for combination. After completion of phase Ia part, one or two selected dose regimens would be expanded to explore the preliminary of anti‐tumor activity of BC001 alone or combination with paclitaxel for patients with selected tumors.

In dose escalation part, all patients would be administrated BC001. In dose expansion part, all patients would be infused BC001 and paclitaxel. BC001 was given as intravenous infusion for no less than 1 h. While, paclitaxel was intravenous infused about 1 h. The treatment could be continued until disease progression, unacceptable toxicity, initiation of other anti‐tumor treatment, treatment refusion, withdrawal of consent, or the end of the study, whichever occurred first.

### 2.2 Patients

Patients (aged ≥18 years‐old) with refractory pathological confirmed advanced solid tumors with measurable lesions (as defined by RECIST V1.1, for phase Ia part) or target lesions (as defined by RECIST V1.1, for phase Ib), an ECOG ≤1, adequate organ functions and life expectancy ≥3 months, only first‐line treatment failure (only for phase Ib), toxicities with prior anti‐tumor therapy recover to CTCAE ≤ grade 1 (NCI‐CTCAE v5.0) or baseline (except any grade alopecia and skin pigmentation) were eligible for enrollment. Patients were excluded if prior anti‐VEGF/VEGFR therapy within 4 weeks, second malignant tumors except for cured locally cancers within 3 years, basal or squamous cell skin cancer, or carcinoma in situ of the cervix; meningeal or symptomatic central nervous system metastases, active infection; a history of ≥ grade 3 gastrointestinal perforation or bleeding within 3 months; a history of vaccine, anti‐tumor therapy, surgery within 4 weeks before the study; a history of colony stimulating factor or erythropoietin, serious gastrointestinal disease within 2 weeks before the study, allergy to study drug; a history of immunodeficiency diseases, organ transplantation, hepatitis B (HBV‐DNA > 10^3^ copies/mL), hepatitis C, syphilis, or HIV infection, immunodeficiency diseases, organ transplantation; alcohol or drug abuse, uncontrolled cardiac disease and infection, or continuous use of NSAIDs.

### 2.3 Endpoints

In phase Ia study, the primary endpoints were to assessed the Dose limited toxicity (DLT), maximum tolerated dose (MTD), recommended dose for expansion (RDE) and safety. The secondary endpoints included pharmacokinetics (PK) profile, objective response rate (ORR), disease control rate (DCR), duration of response (DoR) and progression free survival (PFS). In phase Ib study, the primary endpoint aimed to assess the primary efficacy data including ORR, DCR, DoR and PFS. The secondary endpoint was to assess the safety profile.

### 2.4 Safety assessment

Safety assessment was across the entire study. All adverse events were recorded and categorized according to severity (NCI‐CTCAE V.5.0) and relationship with BC001 or chemotherapy. Dose limiting toxicity (DLT) was defined as the treatment related adverse events (TRAEs) met the DLT criteria during the first 28‐day tolerability observation. The maximum tolerance dose (MTD) was defined as the dose below the dose level at which two patients experienced a DLT.

DLT criteria included grade 3 transaminase escalation duration >7 days, grade 3 hypertension cannot relieve to grade 2 with blood pressure medications for 7 days, grade 2 proteinuria lasting >7 days, grade 3 gastrointestinal reaction with support care, any other ≥3 non‐hematological toxicities. Hematological toxicities meeting DLT criteria included grade 3 febrile neutropenia or grade 4 neutropenia duration >3 days, grade 3 thrombocytopenia for 5 days or with bleeding symptoms, and other grade 4 hematological toxicities.

### 2.5 Pharmacokinetics and pharmacodynamics assessments

The PK parameters were estimated using non‐compartmental analysis (Phoenix WinNonlin 8.1, Certara, Princeton, NJ). The PK parameters of multiple doses were adopted trough concentration for drug accumulation analysis. The serum con‐centration of BC001 was determined by a validated electrochemical luminescence assay. Blood samples were collected at pre‐dose (0.5 h before administration) and post‐dose (2 min, 0.5, 1, 1.5, 2, 2.5, 4, 8, 24, 48, 96, 168, 264, 336 h after the end of infusion) for cycle 1; and at pre‐dose and post‐dose (2 min after the end of infusion) for day 29 of cycle 1.

Circulating concentrations of VEGF‐A, sVEGFR‐1, and sVEGFR‐2 were measured from blood samples obtained at pre‐dose and post‐dose (1, 4, 8, 24, 48, 96, 168 h after the first dose of BC001) for cycle 1; and at pre‐dose and post‐dose for day 15, 29 of cycle 1.

### 2.6 Anti‐tumor activity assessment

Imaging assessments were performed at screening and every 8 weeks. The response was evaluated by RECIST 1.1 for solid tumors. In case of partial response (PR) or better effectiveness, the response should be confirmed after 4 weeks.

### 2.7 Statistical analysis

Statistical analysis was performed by SAS 9.4 (SAS Institute, Cary, NY, USA). The safety assessment was conducted in patients who received at least one dose of BC001 and finished at least one safety assessment. The efficacy was analyzed in patients who completed at least one tumor evaluation. The Clopper‐Pearson method was used to determine the objective response (ORR), disease control rate (DCR) and the exact 95% confidence interval (CI). The Kaplan–Meier method was used to estimate PFS, DoR, OS, with the 95% CI of median time. Data for analysis of phase Ia and Ib were collected up to 20th March 2022 and 21st October 2022, respectively.

## 3 RESULTS

### 3.1 Patient characteristics

**FIGURE 1:**
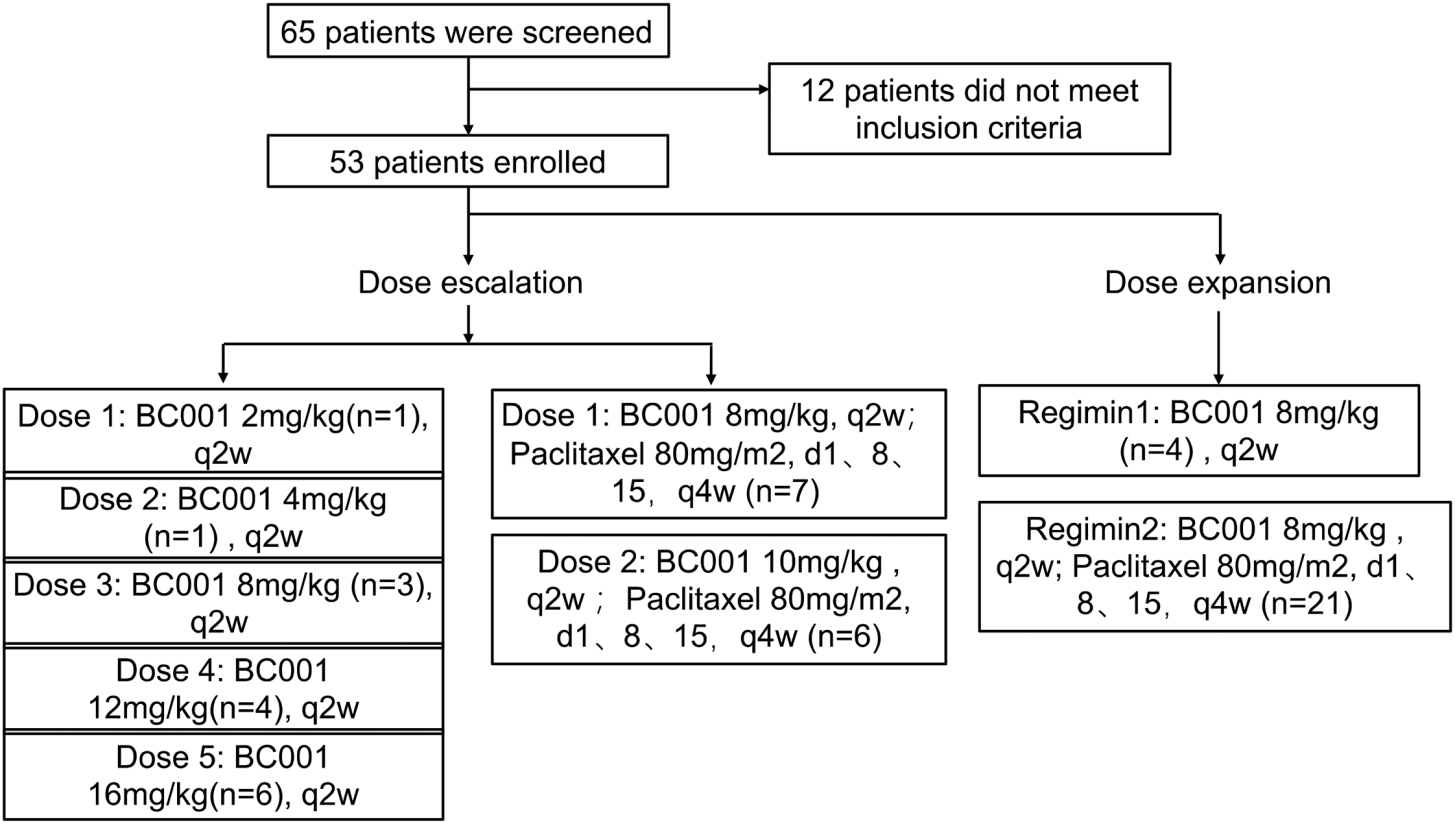
Patient disposition.

From 21st Feb. 2019, a total of 65 patients (phase Ia, *n* = 36; phase Ib, *n* = 29) were screened for this study. Finally, 53 patients were enrolled (phase Ia, *n* = 28; phase Ib, *n* = 25). The last patient enrolled at 21st Apr. 2022. In phase Ia part, 15 patients received BC001 alone at one of five doses [2 mg/kg (*n* = 1), 4 mg/kg (*n* = 1), 8 mg/kg (*n* = 3), 12 mg/kg (*n* = 4), 16 mg/kg (*n* = 6)]. Another 13 patients received BC001 at dose of 8 mg/kg (*n* = 7) or 10 mg/kg (*n* = 6) combination with paclitaxel. In phase Ib part, 25 gastroesophageal junction (GEJ) and gastric cancer (GC) patients received BC001 alone (*n* = 4) or combination with paclitaxel (*n* = 21) at dose level of 8 mg/kg to explore the efficacy. The BC001 single‐agent cohort was early finished for difficult enrollment (Figure 1).

The patients' characteristics were shown in Table 1. Majority of patients were males (*n* = 41, 77.4%), with at least 1‐line prior therapy of chemotherapy, radiotherapy, immunotherapy, targeted therapy and so on. Most patients (*n* = 36, 67.9%) suffered gastric cancer. Nearly a quarter of patients (*n* = 13, 24.5%) experienced tumor lesions resection. Only a small part of patients (*n* = 4, 7.5%) received prior immunotherapy of mono‐antibodies or bio‐specific antibodies targeting PD‐1/PD‐L1, CTLA4 and TGF‐βRII.

**TABLE 1:** Patient demographics and baseline clinical characteristics (FAS).

Characteristics	Phase Ia (*n* = 28)		Phase Ib (*n* = 25)		Total (*n* = 53)
	BC001 (*n* = 15)	BC001 + PTX (*n* = 13)	BC001 (*n* = 4)	BC001 + PTX (*n* = 21)	
Age (Median years, range)	53.0 (37.0–74.0)	57.0 (29.0–76.0)	49.0 (45.0–68.0)	61.0 (31.0–73.0)	57.0 (29.0–76.0)
Sex					
Male	13 (86.7%)	11 (84.6%)	3 (75.0%)	14 (66.7%)	41 (77.4%)
Female	2 (13.3)	2 (15.4%)	1 (25.0%)	7 (33.3%)	12 (22.6%)
ECOG					
0	2 (14.3%)	3 (23.1%)	1 (25.0%)	6 (28.6%)	12 (22.6%)
1	13 (86.7%)	10 (76.9%)	3 (75.0%)	15 (71.4%)	41 (77.4%)
No. of organs of metastasis					
0	0	1 (7.7%)	0	1 (4.8%)	2 (3.8%)
1	7 (46.7%)	8 (61.5%)	0	2 (9.5%)	17 (32.1%)
2	4 (26.7%)	4 (30.8%)	4 (100%)	8 (38.1%)	20 (37.7%)
3	2 (13.3%)	0 (0%)	0	7 (33.3%)	9 (17.0%)
≥4	2 (13.3%)	0 (0%)	0	3 (14.3%)	5 (9.4%)
Tumor types					
Non‐Hodgkin lymphoma	2 (13.3%)	0	0	0	2 (3.8%)
GEJ/Gastric cancer	2 (13.3%)	9 (69.2%)	4 (100.0%)	21 (100.0%)	36 (67.9%)
Colorectal cancer	8 (53.3%)	1 (7.7%)	0	0	9 (16.9%)
Cholangiocarcinoma	1 (6.7%)	0	0	0	1 (1.9%)
Neuroendocrine tumor	1 (6.7%)	1 (7.7%)	0	0	2 (3.8%)
Pancreatic cancer	0	1 (7.7%)	0	0	1 (1.9%)
Duodenal cancer	0	1 (7.7%)	0	0	1 (1.9%)
ESCC	1 (6.7%)	0	0	0	1 (1.9%)
Lines of prior anticancer therapies					
1	1 (6.6%)	7 (53.8%)	4 (100.0%)	21 (100.0%)	33 (62.3%)
2	4 (26.7%)	4 (30.8%)	0	0	8 (15.1%)
≥3	10 (66.7%)	2 (15.4%)	0	0	12 (22.6%)
Prior therapies					
Surgery	4 (26.7%)	5 (38.5%)	3 (75.0%)	1 (4.8%)	13 (24.5%)
Radiotherapy	4 (26.7%)	3 (23.1%)	0	1 (4.8%)	8 (15.1%)
Chemotherapy	15 (100.0%)	13 (100.0%)	3 (75.0%)	21 (100.0%)	52 (98.1%)
Immunotherapy	2 (14.3%)	2 (15.4%)	0	0	4 (7.5%)
Target therapy	3 (20.0%)	1 (7.7%)	1 (25.0%)	1 (4.8%)	5 (9.4%)
Others	4 (26.7%)	1 (7.7%)	1 (25.0%)	1 (4.8%)	7 (13.2%)

Abbreviation: ESCC, esophageal squamous cell carcinoma.

### 3.2 Safety

A total of 53 patients were included in safety assessment. One DLT (grade 4 neutropenia lasting 4 days) was observed and another 1 patient also suffered grade 4 neutropenia, but not met the criteria of DLT in cohort of BC001(10 mg/kg) combination with paclitaxel. Maximum tolerated dose (MTD) was not reached.

All treatment‐emergent adverse events (TEAEs) of BC001 were shown in Table 2. All patients suffered TEAEs of any grade and majority of them (*n* = 46, 86.8%) related to study drug. Twenty‐four of them (45.3%) experienced ≥ grade 3 TEAEs. There were 7 patients (13.2%) experienced treatment‐emergent serious adverse events (TESAE), but only one of them suffered drug related TESAE. During the study, 7 patients dropped out for TEAEs and 3 patients dropped out for drug related TEAE. None of them was dead for drug related TEAEs.

**TABLE 2:** Treatment‐emergent adverse events.

	BC001 single‐agent		BC001 combination with paclitaxel		Total (*n* = 53)
	Phase Ia (*n* = 15)	Phase Ib (*n* = 4)	Phase Ia (*n* = 13)	Phase Ib (*n* = 21)	
Any TEAE	15 (100.0%)	4 (100.0%)	13 (100.0%)	21 (100.0%)	53 (100.0%)
≥G3 TEAE	3 (20.0%)	1 (25.0%)	7 (53.8%)	13 (61.9)	24 (45.3%)
TESAE	0	0	3 (23.1%)	4 (19.0%)	7 (13.2%)
TEAE leading to drop out	1 (6.7%)	0	3 (23.1%)	3 (14.3%)	7 (13.2%)
TRAE	14 (93.3%)	2 (50.0%)	12 (92.3%)	18 (85.7%)	46 (86.8%)
TRSAE	0	0	0	1 (4.8%)	1 (1.9%)
TRAE leading to drop out	0	0	1 (7.7%)	2 (9.5%)	3 (5.7)
TRAE leading to death	0	0	0	0	0
DLT	0	‐	1 (7.7%)	‐	1 (1.9%)
TEAE occurring in ≥3 patients					
Leukopenia	6 (40.0%)	0	8 (61.5%)	19 (76.2%)	33 (62.3%)
Hemoglobin decreased	6 (40.0%)	1 (25.0%)	8 (61.5%)	17 (81.0%)	32 (60.4%)
Neutropenia	5 (33.3%)	0	7 (53.8%)	17 (81.0%)	29 (54.7%)
Elevated TBIL	8 (53.3%)	0	3 (23.1%)	10 (47.6%)	21 (39.6%)
Proteinuria	9 (60.0%)	1 (25.0%)	1 (7.7%)	9 (42.9%)	20 (37.7%)
Hemangioma	7 (46.7%)	0	3 (23.1%)	9 (42.9%)	19 (35.8%)
Alopecia	0	0	6 (38.5%)	9 (45.0%)	15 (28.3%)
Elevated AST	4 (26.7%)	0	5 (38.5%)	6 (28.6%)	15 (28.3%)
Hypoalbuminemia	5 (33.4%)	0	2 (15.4%)	6 (28.6%)	13 (24.5%)
Elevated ALT	4 (26.7%)	0	3 (23.1%)	6 (28.6%)	13 (24.5%)
Peripheral neurotoxicity	0	0	3 (23.1%)	9 (42.9%)	12 (22.6%)
Thrombocytopenia	5 (33.3%)	0	0	6 (28.6%)	11 (20.8%)
Hyperglycemia	1 (13.4%)	1	1 (7.7%)	8 (35.0%)	11 (20.8%)
Hyponatremia	0	1 (25.0%)	0	9 (42.9%)	10 (18.9%)
Nausea	1 (6.7%)	0	3 (23.1%)	6 (28.6%)	10 (18.9%)
Weight loss	1 (6.7%)	1 (25.0%)	3 (23.1%)	5 (23.8%)	10 (18.9%)
Hypokalemia	1 (13.3%)	0	0	8 (38.1%)	9 (17.0%)
Diarrhea	0	0	1 (7.7%)	6 (28.6%)	7 (13.2%)
Fatigue	1 (6.7%)	0	0	6 (28.6%)	7 (13.2%)
Limb pain	0	0	2 (15.4%)	4 (19.0%)	6 (11.3%)
Hypertension	2 (13.3%)	0	2 (15.4%)	2 (9.5%)	6 (9.4%)
Fever	1 (6.7%)	0	2 (15.4%)	2 (9.5%)	5 (9.4%)
Vomiting	0	0	1 (7.7%)	4 (19.0%)	5 (9.4%)
Elevated DBIL	5 (33.3%)	0	0	0 (45.0%)	5 (9.4%)
Anorexia	1 (6.7%)	0	2 (15.4%)	1 (4.8%)	4 (7.5%)
Constipation	2 (13.3%)	0	1 (7.7%)	2 (9.5%)	4 (7.5%)
Elevated IBIL	2 (13.3%)	0	2 (15.4%)	0	4 (7.5%)
Hypocalcemia	0	0	0	4 (19.0%)	4 (7.5%)
Gain weight	0	0	1 (7.7%)	3 (14.3%)	4 (7.5%)
Lymphopenia	3 (20.0%)	0	0	0	3 (5.7%)
Abdominal pain	1 (6.7%)	1 (25.0%)	1 (7.7%)	0	3 (5.7%)
Decreased total protein	0	0	0	3 (14.3%)	3 (5.7%)
Dizzy	0	0	1 (7.7%)	2 (9.5%)	3 (5.7%)
Grade ≥3 TEAE					
Neutropenia	0	0	3 (23.1%)	10 (47.6%)	13 (24.5%)
Leukopenia	0	0	2 (15.4%)	8 (38.1%)	10 (18.9%)
Hemoglobin decreased	0	1 (25.0%)	0	3 (14.3%)	4 (7.5%)
Hyponatremia	0	1 (25.0%)	0	2 (9.5%)	3 (5.7%)
Elevated TBIL	1	0	0	2 (9.5%)	3 (5.7%)
Hypertension	1	0	2 (15.4%)	0	3 (5.7%)
Hypokalemia	1	0	0	1 (5.0%)	2 (3.8%)
Biliary tract infection	0	0	1 (7.7%)	0	1 (1.9%)
Hyperglycemia	1 (6.7%)	0	0	0	1 (1.9%)
Elevated DBIL	1 (6.7%)	0	0	0	1 (1.9%)
Elevated TBIL	1 (6.7%)	0	0	0	1 (1.9%)
Intracranial hemorrhage	0	0	1 (7.7%)	0	1 (1.9%)
ARDS	0	0	1 (7.7%)	0	1 (1.9%)
Interstitial lung disease	0	0	1 (7.7%)	0	1 (1.9%)
Gastrointestinal perforation	0	0	0	1 (5.0%)	1 (1.9%)
Diarrhea	0	0	1 (7.7%)	0	1 (1.9%)
Heart failure	0	0	1 (7.7%)	0	1 (1.9%)

Abbreviations: ALP, alkaline phosphatase;ALT, alanine aminotransferase; ARDS, acute respiratory distress syndrome; AST, Aspartate aminotransferase; CK, creatine kinase; DBIL, conjugated bilirubin; IBIL unconjugated bilirubin; TBIL, total bilirubin; TEAE, treatment‐emergent adverse event.

More than half of patients experienced hematological toxicities, including leukopenia (*n* = 33, 62.3%), hemoglobin decreased (*n* = 32, 60.4%), neutropenia (*n* = 29, 54.7%) and thrombocytopenia (*n* = 11, 20.8%). Elevated TBIL (*n* = 21, 39.6%), proteinuria (*n* = 20, 37.7%), hemangioma (*n* = 19, 35.8%) were the most common non‐hematological toxicities. Neutropenia, leukopenia, hemoglobin decreased were the most commonly observed as ≥ grade 3 hematological TEAEs. While, hyponatremia, elevated TBIL and hypertension were the most common ≥ grade 3 non‐hematological TEAEs. Of note, one gastric patient receiving BC001 (8 mg/kg) in combination with paclitaxel in phase Ib part suffered gastric perforation, which considered as drug‐related TESAE, after 2 doses of BC001 infusion. This patient finally recovered after gastric cancer lesion resection. Another 1 patient suffered intracranial hemorrhage, which might relate to disease progression of new emerged brain metastatic lesions and not relate to BC001. Furthermore, the only 1 patient suffered interstitial lung disease and ARDS related to disease progression.

### 3.3 Pharmacokinetics and pharmacodynamics characteristics

**FIGURE 2:**
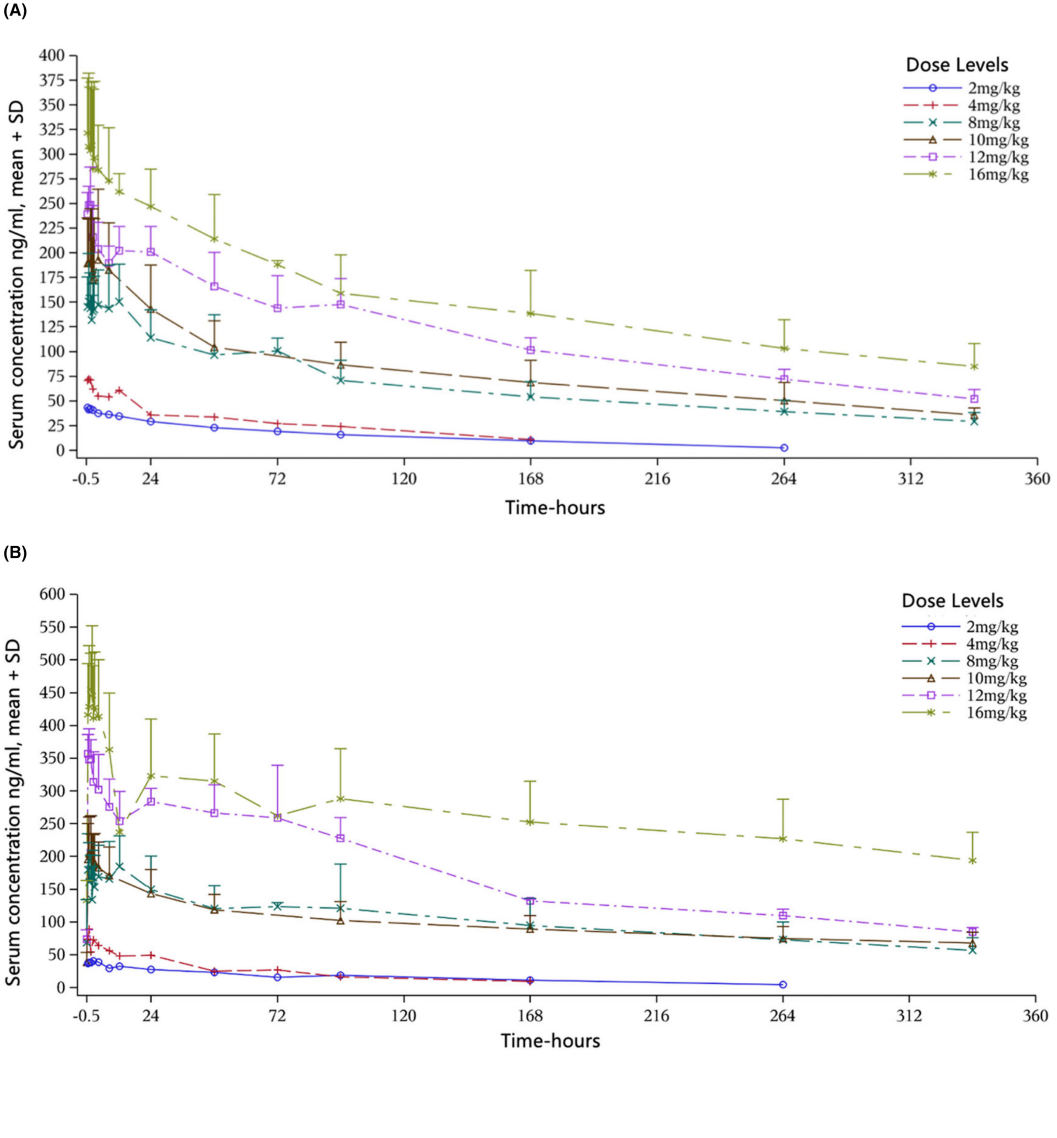
Mean concentration‐time curve of serum BC001. (A) Mean concentration‐time curve of serum BC001 after first infusion. (B) Mean concentration‐time curve of serum BC001 after multiple doses.

All patients (*n* = 28) in phase Ia part received PK and PD evaluation. The serum concentrations and PK data of BC001 were shown in Table 3 and Figure 2. After the first and repeated BC001 infusion, the AUC_0‐t_, AUC_0‐∞_, AUC_τ‐ss_ and *C*_max_, *C*_max_ss_, *C*_min_ss_, were accelerated with dose levels. After combination with paclitaxel, the PK parameters were not largely influenced.

**TABLE 3:** Pharmacokinetics (PK) parameters in different dose level of phase Ia part.

Parameter	BC001 single‐agent															BC001 + paclitaxel					
	2 mg/kg (*N* = 1)			4 mg/kg (*N* = 1)			8 mg/kg (*N* = 3)			12 mg/kg (*N* = 4)			16 mg/kg (*N* = 6)			8 mg/kg (*N* = 7)			10 mg/kg (*N* = 6)		
	*n*	Mean	SD	*n*	Mean	SD	*n*	Mean	SD	*n*	Mean	SD	*n*	Mean	SD	*n*	Mean	SD	*n*	Mean	SD
Single dose																					
*T*_max_ (h)	1	1.02	‐	1	1.53	‐	3	4.21	4.1538	4	1.528	0.422	6	2.235	1.4942	7	3.333	1.9058	6	2.477	1.9766
T_1/2_ (h)	1	69.8	‐	1	74.3	‐	3	177.0	44.58	4	143.4	38.052	6	238.5	66.43	7	165.3	40.1	6	241.5	46.28
*C*_max_(ug/ml)	1	43.1	‐	1	72.1	‐	3	218.0	56.82	4	265.3	23.41	6	335.0	62.95	7	150.9	35.13	6	209.0	65.75
AUC_0‐t_ (h × μg/ml)	1	3380.0	‐	1	4760.0	‐	3	26500.0	4557.41	4	32100.0	14845.65	6	50066.7	11855.24	7	17262.9	6311.23	6	23833.3	5935.21
AUC_0‐∞_(h × μg/ml)	1	4150.0	‐	1	5940.0	‐	3	36133.3	6921.22	4	44825.0	14247.89	6	78600.0	18301.91	7	24371.4	7557.1	6	42100.0	14643.5
AUC__%Extrap_ (%)	1	6.29	‐	1	19.9	‐	3	26.07	8.262	4	32.13	17.613	6	36.22	7.248	7	29.91	14.106	6	41.18	12.0
CL/F (mL/h/kg)	1	0.482	‐	1	0.674	‐	3	0.2273	0.0476	4	0.2973	0.1272	6	0.2118	0.0429	7	0.3559	0.1045	6	0.2567	0.0679
*V*_d_ (mL)	1	48.6	‐	1	72.3	‐	3	56.77	10.257	4	56.65	7.295	6	74.33	33.278	7	81.56	21.696	6	88.10	25.993
λz (1/h)	1	0.0099	‐	1	0.0093	‐	3	0.0041	0.0011	4	0.0052	0.0017	6	0.0031	0.0008	7	0.0044	0.001	6	0.0029	0.0006
MRT_0‐t_ (h)	1	81.7	‐	1	60.9	‐	3	124.3	4.16	4	102.5	47.05	6	133.3	4.41	7	110.47	30.585	6	114.27	23.35
MRT_0‐∞_ (h)	1	99.5	‐	1	104.0	‐	3	249.7	58.71	4	205.5	54.66	6	337.0	73.94	7	231.1	49.34	6	332.5	54.39
Multiple dose																					
*C*_max‐ss_ (μg/ml)	1	40.4	‐	1	89.0	‐	3	228.0	55.05	3	373.7	32.32	6	489.5	78.04	5	179.6	52.61	4	221.5	55.87
*C*_min‐ss_ (μg/ml)	1	0	‐	1	0	‐	3	62.23	25.259	3	73.80	14.155	6	133.2	30.29	5	38.32	15.114	4	38.98	15.033
AUC_τ‐ss_ (h × μg/ml)	1	4360.0	‐	1	4960.0	‐	3	41933.3	12708.0	3	55233.3	5419.72	6	88583.3	19791.66	5	27800.0	9427.09	4	32375.0	7463.41
*T*_max‐ss_ (h)	1	3.03	‐	1	1.5	‐	3	2.40	1.1439	3	1.857	0.2926	6	2.047	0.4367	5	2.192	1.5811	4	2.150	0.8841
λz (1/h)	1	0.0087	‐	1	0.0117	‐	3	0.0032	0.0015	3	0.0042	0.0018	6	0.002	0.0005	5	0.0027	0.0003	4	0.0019	0.0007
*T*_1/2_ (h)	1	79.3	‐	1	59.4	‐	3	245.7	102.01	3	183.3	65.32	6	372.5	102.83	5	262.0	27.17	4	432.0	203.94
*C*_av‐ss_ (ug/ml)	1	13.0	‐	1	14.8	‐	3	125.0	38.12	3	164.3	16.04	6	263.7	59.27	5	82.68	27.903	4	96.50	22.235

Abbreviations: AUC__%Extrap_, area under the concentration‐ time cure derived after extrapolation of % residual; AUC_0–∞_, area under the concentration–time curve from zero extrapolated to infinity; AUC_0–t_, area under the concentration‐time curve from zero to the last time point measured; AUC_τ‐ss_, area under the curve during the dosing interval at steady state; *C*_av‐ss_, average steady state concentration; CL/F, bioavailability clearance; *C*_max_, maximum concentration; *C*_max‐ss_, maximum concentration at steady state; *C*_min‐ss_, minimum concentration at steady state; h, hours; MRT_0‐∞_, mean residence time–time from zero extrapolated to infinity; MRT_0‐t_, mean residence time–time from zero to the last time point measured; *T*_1/2_, half‐life; *T*_max_, time to reach the maximal concentration; *V*_d_, apparent volume of distribution; λz, elimination rate constant.

The concentration of VEGF‐A, sVEGFR‐1, and sVEGFR‐2 after the first dose and multiple doses infusion were shown in Figures S3‐S5. Serum VEGF‐A concentrations increased almost immediately after treatment in all patients. And it remained elevated about 1.3–2.5‐fold from baseline at 7 days. sVEGFR‐1 showed remained relatively constant although some patients exhibited elevated levels. sVEGFR‐2 showed slightly decrease during the 8 h after first infusion and generally recovering to pretreatment levels or slightly increase within 1 day. For all serum biomarkers, the observed changes in levels did not appear to be dose‐related or affected by combination with paclitaxel.

### 3.4 Immunogenicity

There were 27 patients in phase Ia part (single agent cohort: *n* = 17, combination with chemotherapy cohort: *n* = 12) finished immunogenicity evaluation. Only 2 patients had positive serum ADA on the baseline. But after BC001 infusion, the serum ADA was negative. The serum ADA of the rest 25 patients was all negative.

### 3.5 Dose determination

In dose escalation part (phase Ia), no DLT and MTD were observed at all dose levels of single‐agent cohort. Only 1 DLT was observed at dose level of 10 mg/kg of combination cohort. In general, the safety profile was acceptable. Although, there was no patient achieved objective response, more patients (*n* = 3) achieved stable disease at dose level of 8 mg/kg. Meanwhile, in the combination cohort of phase Ia part, the DCR at dose level of 8 mg/kg also seemed higher than that at dose level of 10 mg/kg (87.5% vs. 50.0%), as well as median PFS (5.3 vs. 4.8 months) (Table 4). Thus, based on the data of safety, anti‐tumor acitivity, PK and ADA data, the recommended dose for expansion (RDE) of BC001 was 8 mg/kg.

**TABLE 4:** The anti‐tumor evaluation for patients in this phase Ia/Ib study.

Response	BC001 single‐agent						BC001 combination with paclitaxel		
	Phase Ia					Phase Ib	Phase Ia		Phase Ib
	2 mg/kg (*N* = 1)	4 mg/kg (*N* = 1)	8 mg/kg (*N* = 3)	12 mg/kg (*N* = 4)	16 mg/kg (*N* = 6)	8 mg/kg (*N* = 4)	8 mg/kg (*N* = 7)	10 mg/kg (*N* = 6)	8 mg/kg (*N* = 21)
CR	0	0	0	0	0	0	0	0	0
PR	0	0	0	0	0	0	0	0	6 (28.6%)
SD	1 (100.0%)	0	3 (100.0%)	0	1 (16.7%)	0	6 (85.7%)	3 (50.0%)	10 (47.6%)
PD	0	1 (100.0%)	0	2 (50.0%)	5 (83.3%)	3 (75.0%)	0	1 (16.7%)	4 (19.0%)
NA	0	0	0	2 (50.0%)	0	1 (25.0%)	1 (14.3%)	2 (33.3%)	1 (4.8%)
ORR	0	0	0	0	0	0	0	0	28.6%
DCR	100.0%	0	100.0%	0	16.7%	0	85.7%	50.0%	76.2%
PFS, months, (95% CI)	NE	NE	NE	NE	NE	1.4 (0.9, NA)	5.26 (2.4, NA)	4.75 (1.4, NA)	5.4 (1.9, 7.0)
DoR, months, (95% CI)	NE	NE	NE	NE	NE	NA	0	NE	5.1 (2.6, NA)
OS, months, (95% CI)	NE	NE	NE	NE	NE	3.1 (1.5, NA)	NE	NE	9.4 (5.4, NA)

Abbreviations: CR, complete response; DCR, disease control rate; NA, non‐acquired; NA, not acquired; NE, non‐evaluable; ORR, objective response rate; PD, progressive disease; PR, partial response; SD, stable disease.

### 3.6 Anti‐tumor activity

**FIGURE 3:**
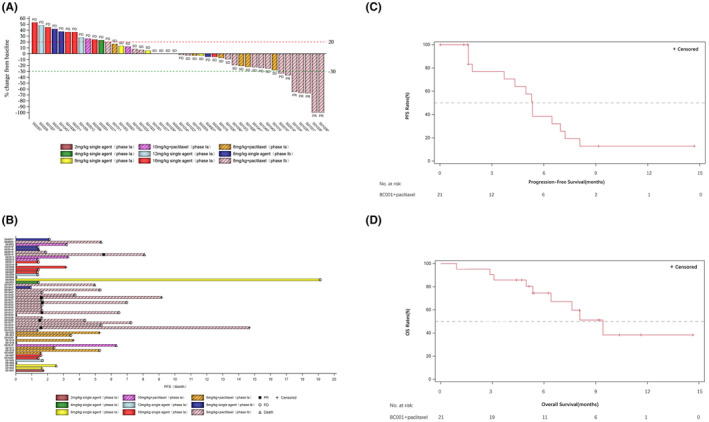
Response, duration of treatment, and survival analysis. (A) Response and changes of targeted lesions in phase Ia/Ib. Patients with GC/GEJ were marked with “*”. (B) Outcome and duration of BC001 in phase Ia/Ib. Patients with GC/GEJ were marked with “*”. (C) Progression‐free survival in second‐line treatment of GEJ/GC patients who receiving BC001 plus paclitaxel in phase Ib study(*n* = 21). (D) Overall survival in second‐line treatment of GEJ/GC patients who receiving BC001 plus paclitaxel in phase Ib study(*n* = 21).

A total of 46 patients finished at least 1 efficacy evaluation (phase Ia, *n* = 23; phase Ib, *n* = 23), with 7 patients quitted the study before evaluation for patients' personal choice (phase Ia, *n* = 5; phase Ib, *n* = 2). The efficacy of BC001 alone and combination with paclitaxel was shown in Table 4 and Figure 3A,B.

For patients received BC001 single‐agent, none of them achieved complete response (CR) or PR. There were 5 patients achieved stable disease (SD) (gastric cancer, *n* = 1; non‐Hodgkin lymphoma, *n* = 1; colorectal cancer, *n* = 2; neuroendocrine tumor, *n* = 1), with DCR of 33.3% (5/19). Of note, 1 patient with neuroendocrine tumor (NET) achieved stable disease with duration of 19.1 months after BC001 single‐agent infusion at dose level of 8 mg/kg. In the further study of phase Ib, no one GC patient benefited from BC001 for second‐line treatment and the enrollment was slow. So that, the enrollment of this cohort was early terminated.

For patients who received BC001 combined with paclitaxel, all patients achieving objective response were in the second‐line GEJ/GC treatment in phase Ib study. Among these patients in combination cohort of phase Ib study (*n* = 21), 1 patient withdrew the study before the first efficacy assessment for personal choice up to the last follow‐up. Eleven patients suffered disease progression and 3 patients were dead. There were 6 patients achieved PR, 10 patients experienced SD. The ORR was 28.6% (95% CI 11.3%, 52.2%) and the DCR was 76.2% (95% CI 52.8%, 91.8%), with the median PFS of 5.4 months (95% CI 1.9, 7.0) and OS of 9.4 months (95% CI 5.4, NA) (Figure 3C,D). The median DoR was 5.1 months (95% CI 2.6, NA). Up to the data closed, 3 GC patients who received BC001 in combination with paclitaxel had achieved PFS over 12 months.

## 4 DISCUSSION

BC001, a novel VEGFR2 antibody, could specifically and tightly bind to epitope on the extracellular domain of VEGFR and more completely block VEGFR2/VEGF axis.^9^ This phase Ia/Ib study was conducted to explore the safety, PK/PD profile, and preliminary anti‐tumor activity of BC001 single‐agent and combined with paclitaxel in patients with advanced solid tumors.

MTD was not reached. One DLT (grade 4 neutropenia lasting 4 days) was observed at dose level of BC001 10 mg/kg combination with paclitaxel. No clear dose‐related TEAEs were observed in different dose levels. Nearly half of TEAEs (45.3%) were ≥grade 3, but most of them were observed after BC001 plus paclitaxel infusion. So, the incidence of BC001 contributed ≥ grade 3 TEAEs was very low. And no TRAE related death was observed. The toxicity spectrum was largely similar with another VEGFR‐2 antibody, ramucirumab.^10^, ^11^, ^12^ Compared with ramucirumab, BC001 was seemed to show relative lower incidence of deep venous thrombosis (DVT). No patients experienced DVT after BC001 infusion. Although BC001 showed higher incidence of leukopenia and neutropenia, most of patients experienced grade 1–2 leukopenia and neutropenia. All patients suffering grade 3–4 leukopenia and neutropenia received BC001 combination with paclitaxel, which might be affected by chemotherapy. BC001 also showed lower risk of hypertension with incidence of less than 10%. While 38% of patients showed hypertension after ramucirumab infusion.^13^ Furthermore, more than 30% patients experienced hemangioma, which rarely observed in ramucirumab. It might relate to increased VEGF‐A after BC001 infusion. But none of them suffered BC001 related visceral bleeding. The only 1 patient suffering gastric perforation had a gastric cancer primary lesion without resection. Receiving anti‐VEGR2 treatment might increase the risk of perforation, which also shown in other anti‐VEGF/VEGFR therapies.^14^, ^15^, ^16^, ^17^ In sum, BC001 showed manageable safety profiles.

In the PK analysis, the BC001 PK profile was characterized by dose‐dependent elimination and non‐linear exposure consistent with saturable clearance, which was similar to PK profiles exhibited by ramucirumab.^11^ In cohort of BC001 plus paclitaxel, the exposure and clearance of BC001 was decreased compared with BC001 single‐agent, which indicated that paclitaxel might decrease the exposure and clearance of BC001.

In immunogenicity assessment, 2 patients had positive ADA on the baseline. While after receiving BC001, the ADA was negative, indicating the baseline ADA could be false‐positive. The incidence of ADA was 0% after receiving BC001, which was consistent with ramucirumab.^12^ Generally speaking, BC001 showed low immunogenicity after infusion to patients.

In this study, although no partial response was observed after BC001 infusion, one patient with NET achieved durable stable disease with duration of 19.1 months, indicating that BC001 provides a certain level of clinical benefit and NET might be a novel potential benefit tumor type, which might be further explored. The anti‐tumor activity was comparable with ramucirumab monotherapy as salvage treatment.^18^ Given the low objective response rate of monotherapy, combination therapy is the direction for future exploration. The efficacy of BC001 combination with paclitaxel also explored in second‐line treatment of advanced gastric cancer. The ORR was 28.6% and DCR was 76.2%, with the median PFS of 5.4 months and median OS of 9.4 months. The efficacy and PFS were comparable with ramucirumab and taxanes in second line treatment of gastric cancer.^19^, ^20^, ^21^ The serum levels of VEGF‐A, sVEGFR‐1, sVEGFR‐2 were analyzed during BC001 infusion, but the changes could not predict the efficacy of BC001. Up to now, rare biomarker was found to predict the efficacy of anti‐VEGF/VEGFR therapies.^22^ As a phase I study, the study sample was small to confirm the safety and efficacy of BC001, the results of the study might be limited. Further clinical expansion study of BC001 would be conducted in several other selected tumors, such as NET, colorectal cancer (ChiCTR2300070070), gastric cancer (ChiCTR2300071906) in the future.

In conclusion, BC001 demonstrated favorable safety and clinical response in refractory population of advanced solid tumors.

## AUTHOR CONTRIBUTIONS

**Dan Liu:** Conceptualization (equal); data curation (equal); formal analysis (equal); investigation (equal); project administration (equal); resources (equal); software (equal); supervision (lead); validation (equal); visualization (equal); writing – original draft (lead); writing – review and editing (lead). **Jifang Gong:** Conceptualization (equal); data curation (equal); formal analysis (equal); investigation (equal); project administration (equal); resources (equal); software (equal); supervision (lead); validation (equal); visualization (lead); writing – original draft (supporting); writing – review and editing (lead). **Muling Liu:** Conceptualization (equal); data curation (equal); formal analysis (equal); investigation (equal); project administration (equal); resources (equal); software (equal); supervision (supporting); validation (equal); visualization (equal); writing – original draft (supporting); writing – review and editing (supporting). **Huan Zhou:** Conceptualization (equal); data curation (equal); formal analysis (equal); investigation (equal); project administration (equal); resources (equal); software (equal); validation (equal); visualization (equal). **Shumei Wang:** Conceptualization (equal); data curation (equal); formal analysis (equal); investigation (equal); project administration (equal); resources (equal); software (supporting); validation (equal); visualization (equal). **Jian Yang:** Conceptualization (equal); data curation (equal); formal analysis (equal); investigation (equal); project administration (equal); resources (equal); software (equal); validation (equal); visualization (equal). **Chenwei Shang:** Conceptualization (equal); data curation (equal); formal analysis (equal); investigation (equal); project administration (supporting); resources (supporting); software (supporting); validation (equal); visualization (equal). **Xinlei Guo:** Conceptualization (supporting); data curation (supporting); formal analysis (equal); investigation (supporting); methodology (supporting); project administration (supporting); resources (supporting); software (supporting); supervision (supporting); validation (supporting); visualization (supporting). **Cha Wang:** Conceptualization (supporting); data curation (supporting); formal analysis (supporting); investigation (supporting); project administration (supporting); resources (supporting); software (supporting); validation (supporting); visualization (supporting). **Yanqiao Zhang:** Conceptualization (equal); data curation (equal); formal analysis (equal); funding acquisition (supporting); investigation (equal); methodology (equal); project administration (equal); resources (equal); software (equal); supervision (supporting); validation (equal); visualization (equal); writing – original draft (supporting); writing – review and editing (supporting). **Lin Shen:** Conceptualization (equal); data curation (equal); formal analysis (equal); funding acquisition (lead); investigation (equal); methodology (lead); project administration (equal); resources (equal); software (equal); supervision (lead); validation (equal); visualization (equal); writing – original draft (supporting); writing – review and editing (lead).

## FUNDING INFORMATION

This study was sponsored by Luzhou Buchang Bio‐Pharmaceutical Co., Ltd and supported by the Major Program of National Natural Science Foundation of China (No. 91959205).

## CONFLICT OF INTEREST STATEMENT

Chengwei Shang, Xinlei Guo and Cha Wang were employees of Guangzhou Gloria Biosciences Co., Ltd. Others authors declare that they have no known competing financial interests or personal relationships that could have appeared to influence the work reported in this paper.

## ETHICS STATEMENT

This study was approved by the Ethics Committee of Beijing Cancer Hospital (2018YW75), Ethics Committee of Harbin Medical University Cancer Hospital (2018‐160), Clinical Medicine Research Ethics Committee of the First Affiliated Hospital of Bengbu medical college ([2020]037), and the Clinical Medicine Research Ethics Committee of Cangzhou People's Hospital (2020‐批件‐20).

## Supporting information


Figures S1–S5.

## Data Availability

Reasonable request for data sharing should be submitted to the corresponding author after the indication has been approved in China. The sponsor will review the proposal and consider to share the data providing the requestor signs a data–access agreement.
