# Supplementary material for: Phase I study of BC001, a novel fully human immunoglobulin G1 monoclonal antibody targeting the vascular endothelial growth factor receptor‐2, in advanced solid tumors
Source: Cancer Med. 2024 Nov 19;13(22):e70208. doi: 10.1002/cam4.70208 (PMC11576409; doi:10.1002/cam4.70208)

**Supplementary file**

**Figure S1. The efficacy of BC001 and Ramucirumab in PDX models**

Figure S1a. Effects of BC001 and Ramucirumab on Relative Tumor Volume in Human Gastric Cancer BGC-823 Xenograft Models in Nude Mice

Figure S1b. Effects of BC001 and Ramucirumab on Tumor Weight Distribution in Human Gastric Cancer BGC-823 Xenograft Models in Nude Mice

**Figure S2. Study design**

**
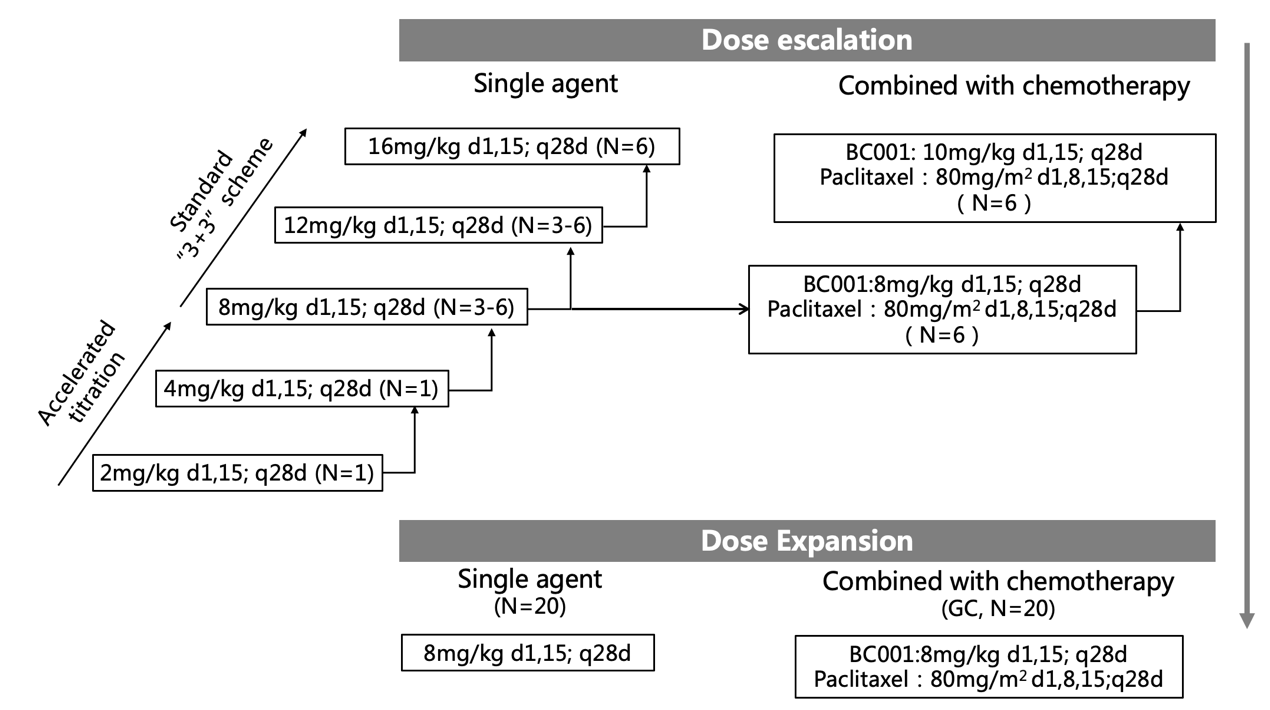
**

**Figure S3. Serum VEGF-A following first dose and multiple doses of BC001**

Figure S3a. Serum VEGF-A following first dose of BC001


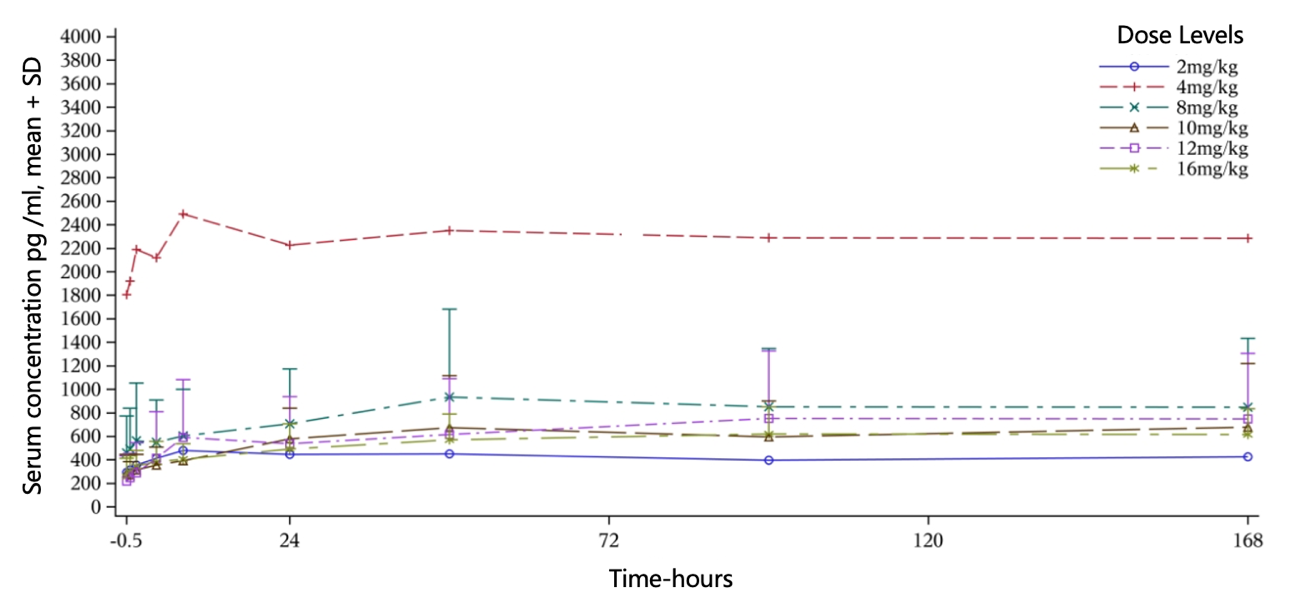


Figure S3b. Serum VEGF-A following multiple doses of BC001


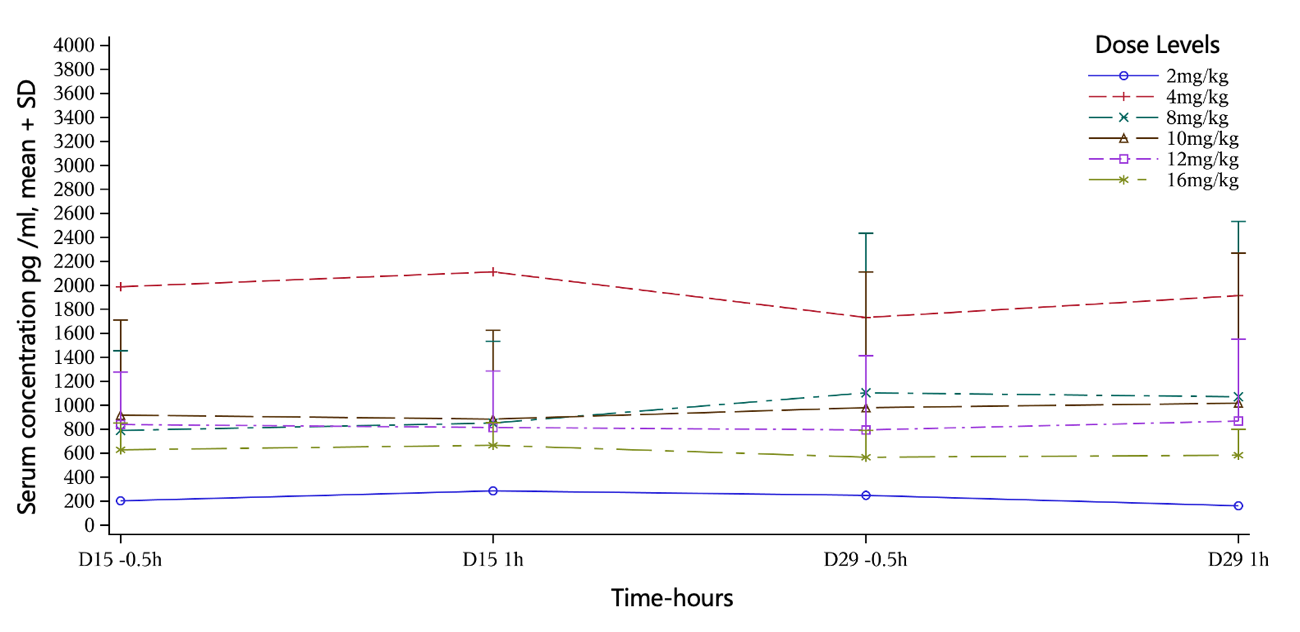


**Figure S4. Serum sVEGFR-1 following first dose and multiple doses of BC001**

Figure S4a. Serum sVEGFR-1 following first dose of BC001


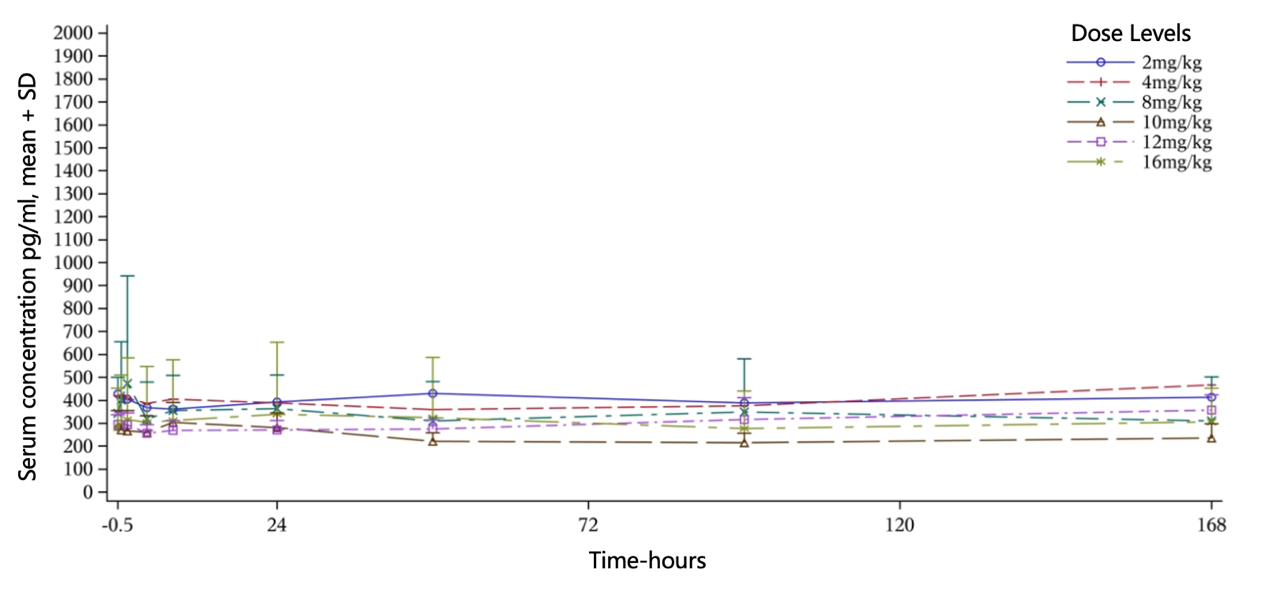


Figure S4b. Serum sVEGFR-1 following multiple doses of BC001


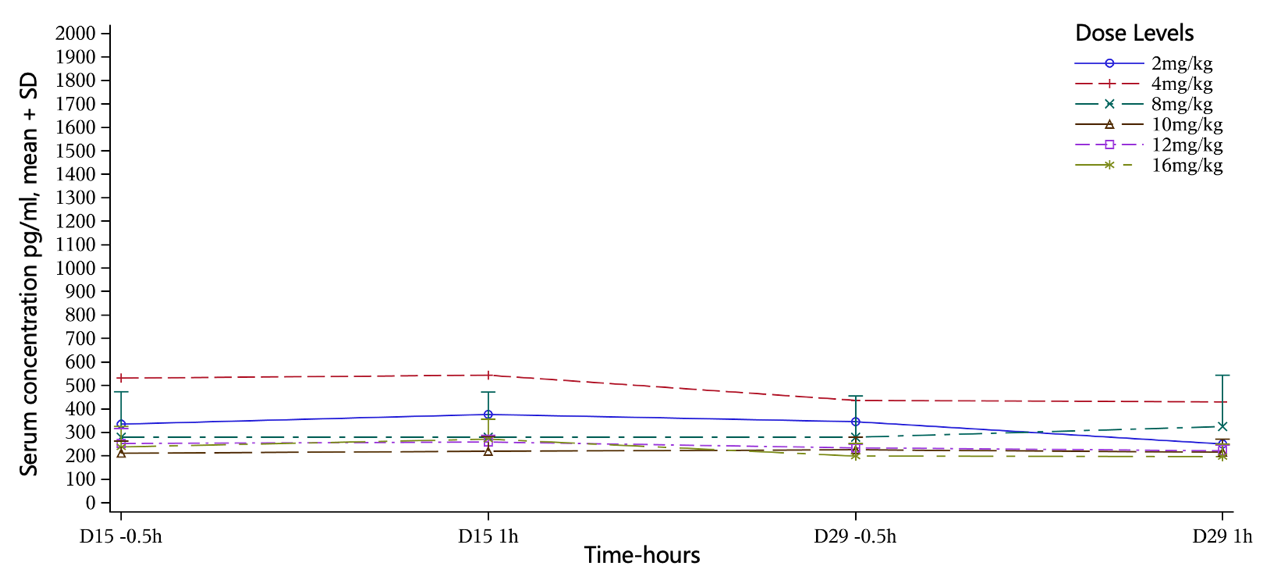


**Figure S5. Serum sVEGFR-2 following first dose and multiple doses of BC001**

Figure S5a. Serum sVEGFR-2 following first dose of BC001


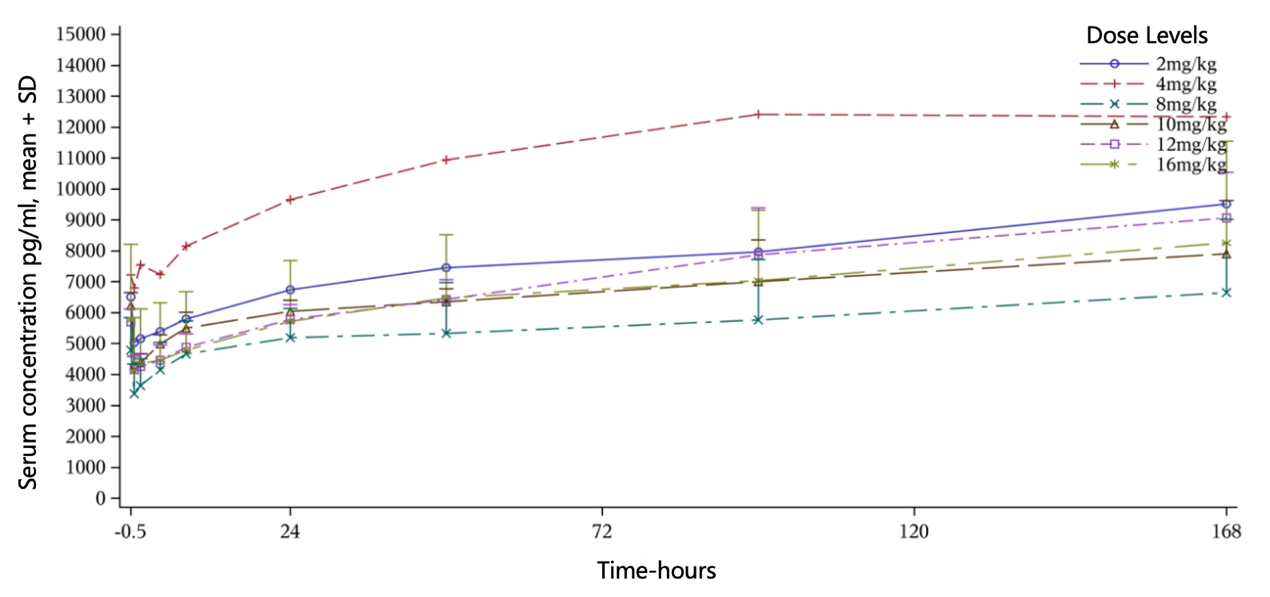


Figure S5b. Serum sVEGFR-2 following multiple doses of BC001


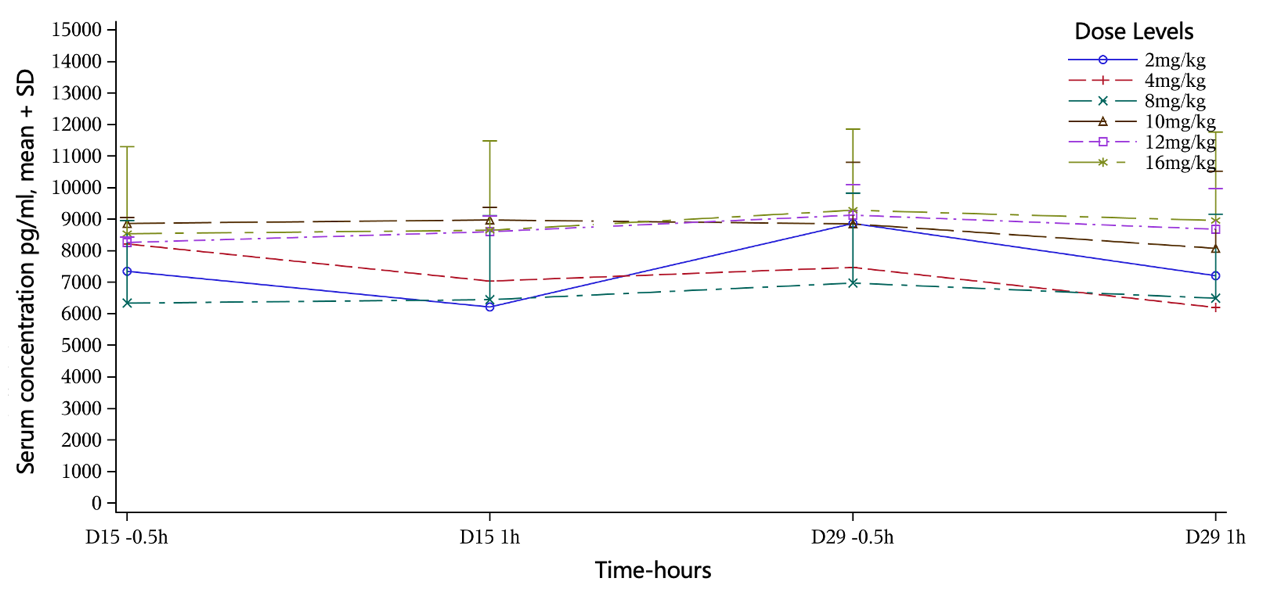

Supplement: Supplementary file 1 — Figures S1–S5. [file CAM4-13-e70208-s001.docx]
